# A Large‐Scale Study on the Relation Between Multilingualism and Social Traits in Adults With and Without Autism Diagnoses

**DOI:** 10.1002/aur.70300

**Published:** 2026-06-23

**Authors:** Margreet Vogelzang, Sean Dong Wei Gan, Jamie Setch

**Affiliations:** ^1^ School of Psychology Newcastle University Newcastle upon Tyne UK; ^2^ Theoretical and Applied Linguistics University of Cambridge Cambridge UK

**Keywords:** autism, bilingualism, communication, multilingualism, social interaction, traits

## Abstract

Autism Spectrum Disorder is a neurodevelopmental condition characterized by persistent difficulties in social interaction and communication alongside restricted, repetitive patterns of behavior, interests, or activities. Emerging evidence suggests that bi/multilingualism may enhance social interaction skills, yet prior research has largely focused on children, relied on small samples, and used oversimplified monolingual–bilingual groupings. The present study examines whether multilingual engagement predicts autism‐associated social traits in adults with and without autism diagnoses. Specifically, we investigated whether childhood and/or current multilingual engagement (operationalized as entropy) predicts social interaction, communication, and overall autistic traits, measured via the Comprehensive Autistic Trait Inventory (CATI). Four hundred and fifty‐nine adults (of which 132 diagnosed autistic) participated in the study. Analyses revealed that, both in the full sample and in the subsample of individuals with autism diagnoses, greater language entropy was associated with *lower CATI scores* in social interaction and overall. No relation between language entropy and communication traits was found. Within the autistic subgroup, the associations between current language entropy and both social interaction and overall CATI scores had large effect sizes (*ηp* ^2^ = 0.14–0.18). These findings show that multilingual engagement is reliably associated with reduced social autistic traits in adulthood.

## Summary

Autism is a developmental condition which often involves social and behavioural difficulties. This study looked at whether speaking more than one language positively affects autistic and no‐autistic adults' social skills. We found that people who regularly use multiple languages showed fewer social difficulties linked to autism. The effect was strongest in autistic adults.

## 1 Introduction

Autism spectrum disorder (ASD) is a neurodevelopmental condition characterized by persistent difficulties in social communication and interaction alongside restricted or repetitive behaviors and interests. Symptoms are heterogeneous and vary in severity but typically emerge early in development and substantially impact daily functioning in autistic individuals. ASD is recognized as a spectrum that includes individuals with diverse abilities and may occur with or without intellectual or language impairments (American Psychiatric Association 2013; World Health Organization 2025).

Recent research with children suggests that speaking multiple languages (bi/multilingualism, henceforth multilingualism) may alleviate some cognitive difficulties associated with ASD (Peristeri, Vogelzang, and Tsimpli 2021; Prévost and Tuller 2022). This aligns with findings in neurotypical populations where multilingualism is linked to cognitive benefits (Adesope et al. 2010; Bialystok 2009; Bialystok et al. 2012; Costa et al. 2008). Here, we explore whether multilingualism might also enhance social interaction traits typically associated with ASD.

### 1.1 Background

ASD affects approximately 1 in 36 children (Maenner et al. 2023) and often co‐occurs with diverse language and cognitive profiles. Simultaneously, multilingualism is increasingly prevalent, with an estimated 60%–70% of the global population using more than one language in daily life (Grosjean 2010). Understanding how multilingual exposure interacts with autistic traits is therefore of growing importance.

Previous research suggests that multilingual effects may stem from its influence on cognitive control processes. In neurotypical populations, multilingualism has been linked to enhanced executive functions (EF), including cognitive flexibility, attentional control, and working memory (Adesope et al. 2010; Bialystok 2009; Costa et al. 2008): the so‐called bilingual advantage. Though the existence of a bilingual advantage in neurotypical populations has been questioned (e.g., Paap and Greenberg 2013; Paap et al. 2015; Samuel et al. 2018), comparable benefits have been reported in autistic populations (Andreou et al. 2020; Peristeri, Vogelzang, and Tsimpli 2021; Romero et al. 2024). This advantage may arise from the continuous need to manage competition between languages (engaging EF), as proposed by the inhibitory control model (Green 1998) and the adaptive control hypothesis (Abutalebi and Green 2007; Green and Abutalebi 2013). Alternatively, it may reflect enhanced attention allocation and regulation, as suggested by the attentional control hypothesis (Bialystok and Craik 2022; Bialystok 2024, 2025).

If a relation between multilingualism and social interaction skills exists, it can occur through at least two distinct pathways. Firstly, multilingualism may directly enhance social skills by exposing individuals to diverse linguistic and cultural contexts that require flexible communication. Alternatively, enhancements may arise indirectly through the bilingual advantage in EF; in this case, enhanced cognitive control and/or Theory of Mind (Andreou et al. 2020; Peristeri, Baldimtsi, et al. 2021) may facilitate social functioning. Regardless of the underlying mechanisms, the first step is to establish whether multilingual exposure is indeed reliably associated with differences in social interaction skills.

Understanding these associations is crucial in applied contexts. Parents of autistic children in multilingual or minority‐language families are frequently advised by clinicians to abandon their heritage language in favor of a monolingual upbringing (Kay‐Raining Bird et al. 2012). This is typically based on concerns that hearing/speaking multiple languages would hinder a child's development, especially when the school language differs from the child's home language (Howard et al. 2020). However, multilingual adults report increased social confidence, communicative flexibility, and cultural belonging, without language or social disadvantages (Nolte et al. 2021). This suggests that abandoning the heritage language may not serve children's best interests and may limit cultural identity development and social opportunities.

Existing research does not support the notion that being raised multilingual negatively impacts social or communication skills in autism. We performed a literature search identifying 23 relevant quantitative, 3 qualitative, and 13 review studies examining social skills at the intersection of autism and multilingualism (see the S1). Two key findings emerged: (i) most studies report positive or neutral impacts of multilingualism on social interaction and communication traits, with very few negative findings; and (ii) most empirical studies (23/26, 88%) focused on children (i.e., individuals aged 0–18).

Most quantitative studies (12/23; 52%) showed no overall effect of multilingualism on social/communication outcomes. For example, multiple studies found no difference in the expressive language abilities of monolingual and multilingual autistic children (Ohashi et al. 2012; Petersen et al. 2012). Reetzke et al. (2015) likewise observed no differences between bilingual and monolingual children, and Wang et al. (2018) concluded that bilingualism has a small or negligible influence on social communication. Similarly, Digard et al. (2023) reported parents overwhelmingly perceived bilingualism as having neutral or positive effects on their child's development, with no evidence of harm. Although findings vary across samples and methodologies, only a small minority of studies (Peristeri et al. 2020; Ratto et al. 2022; Siyambalapitiya et al. 2022; Valicenti‐McDermott et al. 2019) report any negative associations, and these are typically limited to specific measures or subgroups rather than indicating global harm.

Simultaneously, several quantitative studies report advantages for multilingual autistic individuals in communicative and social domains. These include larger vocabularies (Petersen et al. 2012; Hambly and Fombonne 2014), greater production of correct words (Gonzalez‐Barrero and Nadig 2017), greater production of communicative gestures such as pointing, and more adaptive interaction styles (Valicenti‐McDermott et al. 2013; Zhou et al. 2019). Broader benefits have also been observed, including improved social life quality (Digard et al. 2020) and enhanced socialization skills (Hastedt et al. 2023). Qualitative studies reinforce these findings, with autistic multilingual adults reporting enhanced social confidence, cultural integration (Nolte et al. 2021), and richer self‐expression (Digard et al. 2022).

Yet, the existing literature has several limitations. Many studies rely on small samples of often fewer than 100 participants, increasing the risk of statistical errors (Button et al. 2013) and limiting the ability to detect subtle effects. Research has predominantly focused (young) children, meaning the findings may not apply to adults. Perhaps most importantly, 17 of 23 quantitative studies (74%) treated multilingualism as a simple categorical variable (e.g., monolingual vs. bilingual), rather than capturing variation in exposure or frequency of use. Consequently, they overlook differences between multilinguals, such as those who occasionally use a second language and those who engage with multiple languages daily.

Closer examination of a few key studies reveals an interesting pattern. Kašćelan et al. (2019) reported three relevant findings regarding the relationship between bilingualism and autistic‐like traits in children. First, bilingualism did not predict overall autistic‐like traits in the general population. Second, when children were split into high‐ and low‐trait subgroups, an effect emerged only in the high‐trait group, where bilinguals showed lower autistic‐like traits than monolingual peers. Third, their Table 2 showed an advantage for bilinguals on the CCC‐2 Social Relationships subscale (*p* = 0.04), included as part of a broader descriptive overview and not as a primary outcome. Although this effect did not survive correction for multiple comparisons, it is of note for the current investigation. In contrast, they found no group differences in communication.

A similar discrepancy between social interaction and communication can be observed in work by Peristeri and colleagues (Peristeri et al. 2020; Peristeri, Vogelzang, and Tsimpli 2021; Peristeri, Baldimtsi, et al. 2021). In Peristeri et al. (2020), bilingual and monolingual autistic children were matched on autistic traits using the autism Diagnostic Interview‐Revised (ADI‐R; Lord et al. 1994), as the study focused on narrative and executive‐function outcomes rather than autistic traits themselves. However, the ADI‐R descriptives revealed an interesting pattern: bilingual autistic children showed lower (i.e., less impaired) scores in *Reciprocal Social Interaction*, while no group differences emerged in *Communication*. Similar descriptive tendencies appear in Peristeri, Vogelzang, and Tsimpli (2021) and Peristeri, Baldimtsi, et al. (2021), where bilingual autistic children again showed reduced social‐interaction traits but comparable or increased communication traits relative to monolingual peers. The absence of communicative advantages in bilinguals aligns with broader findings that bilingual children may show slightly lower early vocabulary but converge with monolinguals by adulthood (Bialystok 2009; Howard et al. 2024). This suggests that any bilingual advantages beyond cognitive ones are likely social rather than linguistic/communicative in nature.

### 1.2 Present Study

This study aims to address the limitations in the existing literature by employing a dimensional approach that goes beyond simple group comparisons to explore how different facets of language experience relate to social traits associated with autism. The sample includes both autistic and non‐autistic adults, an under‐researched population, allowing for larger‐scale data collection. To explore the relation between multilingualism and social traits, we examine how different measures of multilingualism predict social interaction and communication abilities, as measured by the Comprehensive Autistic Trait Inventory (CATI). We formulated the following research questions (RQs) and hypotheses (Hs):

Does engagement with multiple languages enhance social interaction traits associated with autism?

*Greater engagement with multiple languages (operationalized as* language entropy*, see Sections* *2.3.3* *and* *2.3.4*, *while also testing the effects of age of onset of second language acquisition and second language proficiency, see Section* *2.3.5* *) will be associated with lower scores on the Social Interactions subscale of the CATI*.

Does engagement with multiple languages enhance autistic traits overall?

*Greater engagement with multiple languages will be associated with lower overall autistic traits (i.e., lower full‐scale CATI scores). This effect is expected to be driven by a reduced score on the Social Interactions subscale, with no effect predicted on the Communication subscale. However, engagement with multiple languages could potentially affect scores on the Communications subscale due to its questions relating to Theory of Mind and non‐literal language; thus, this part of the hypothesis is more exploratory*.

Do these effects hold in individuals with an autism diagnosis?

*The association between engagement with multiple languages and autistic traits (overall CATI score, as well as the Social Interactions subscale) will hold within autistic individuals*.

## 2 Methods

### 2.1 Participants

Participants were recruited between August and December 2024 through various channels, namely: (1) designated online recruitment systems: Prolific (https://www.prolific.com/) and Voice (https://voice‐global.org/); (2) social media; (3) mailing lists; (4) Newcastle University School of Psychology's Sona system; and (5) acquaintances. Participants were given gift vouchers, Prolific payments, or Sona credits for their time. A staggered recruitment approach was employed to ensure a balanced mix of multilingual status and autism diagnostic status frequencies.

After screening for invalid and incomplete responses (see Section 2.3.1.), a sample of 459 participants remained. Participants' age in years ranged from 18 to 68 (*M* = 30.46, SD = 11.64). Participants were 253 females, 161 males, and 35 nonbinary or transgender (10 sex/gender unknown). The majority of participants self‐reported to be monolingual (*n* = 258). However, even these participants often reported engagement in multiple languages when probed in more detail. Most participants were ethnically white or European (*n* = 321), Black or African (*n* = 47), Asian (*n* = 43), or mixed (*n* = 30). One hundred and thirty‐two participants reported having been diagnosed with autism, 139 reported suspicions of autism but no formal diagnosis, and 188 participants reported no diagnosis or suspicion (see Section 2.3.2. for details). Other sample demographics are shown in Table 1. The study received ethical approval from Newcastle University's Faculty of Medical Sciences ethics committee (2758/45638).

**TABLE 1:** Sociodemographic characteristics of the participants.

	Total	Monolingual	Bilingual	Multilingual
Total *N* (%)	459 (100)	258 (56)	105 (23)	95 (21)
Age: Mean (SD) [Range]	30.46 (11.64) [18–68]	30.04 (12.66) [18–68]	30.36 (10.55) [18–61]	31.55 (9.73) [18–64]
IMD ranking (*n* = 329) Mean (SD) [Range]	15,508 (10,230) [24–32,843]	17,486 (10,282) [24–32,843]	11,407 (9250) [179–32,725]	13,115 (9360) [117–29,501]
Diagnostic autism status *N* (%)				
Diagnosed	132 (29)	74 (56)	26 (20)	32 (24)
Suspected	139 (30)	83 (60)	28 (20)	28 (20)
No diagnosis or suspicion	188 (41)	101 (54)	51 (27)	35 (19)
Languages known: mean (SD)	2.83 (1.95)	2.30 (1.83)	3.10 (1.72)	3.99 (1.96)

*Note:* The Index of Multiple Deprivation (IMD) is a UK government metric that quantifies area‐level deprivation and serves as a proxy for socioeconomic status (Office for National Statistics 2021).

### 2.2 Materials

Participants completed a Qualtrics survey that gathered information on their demographics, language history, and autistic traits. Additional questionnaires were administered, but these were not relevant to the present investigation. The complete study took approximately 25 min to complete.

#### 2.2.1 Demographic and Language Background Questionnaire

A series of questions were used to gather demographic information, including age, gender, and ethnicity. These were largely taken/adapted from Hughes et al. (2022). We developed a comprehensive language background questionnaire to explore childhood environment and language exposure, current (adulthood) language exposure, and language proficiency. Questions about languages specifically asked about languages/dialects. The questions were largely inspired by the Language Experience and Proficiency Questionnaire (Marian et al. 2007), Bilingual Language Experience Calculator (Unsworth 2013), and Q‐BEx (De Cat et al. 2023). Participants were asked to identify up to 9 individuals they lived with during childhood and the age they started and age they stopped living with each individual. Participants then listed up to 9 languages they had used or been exposed to and when they began acquiring each language. Participants also indicated the percentage of time each of their languages was used when speaking with each previously listed individual. They were also asked to estimate, in their current environments (home, friends, extended family, work, education, and community contexts), the percentage of time currently spent speaking in or exposed to each of their languages while interacting with others.

#### 2.2.2 Comprehensive Autistic Trait Inventory (CATI)

The CATI (English et al. 2021) is a 42‐item autistic trait questionnaire with six subscales of 7 questions each: Social Interactions, Communication, Social Camouflage, Cognitive Rigidity, Repetitive Behaviors, and Sensory Sensitivity. Each item is responded to on a 5‐point likert scale from “Definitely disagree” to “Definitely agree”. We added an “I don't know” option to allow participants to skip items. Five items were reverse scored in line with CATI documentation. A higher score indicates higher levels of autistic traits.

CATI subscale scores were only calculated if participants completed > 70% (i.e., 5 out of 7) of the items. “I don't know” responses were not considered complete items. The overall CATI score was calculated as the average of the subscales and was thus only calculated if all subscales had > 70% completion. Different from the original CATI report (English et al. 2021), we present mean scores (on a scale of 1 to 5) for both the subscales and the overall scale; we deem this to be more interpretable than subscale scores between 7 and 35 and an overall score between 42 and 210.

English et al.'s (2021) first study reported an internal consistency (Cronbach's alpha) of 0.97 for the overall scale and 0.80–0.93 for the subscales. In the present study, the CATI demonstrated an internal consistency of 0.96 for the overall scale and between 0.85 and 0.94 for the subscales, with 0.94 for the Social Interactions subscale (using the *alpha* function from the *psych* package in R, Revelle 2025). These scores evidence good to excellent reliability of the measure. A confirmatory factor analysis was conducted using the *cfa* function from the *lavaan* package (Rosseel 2012) to confirm the presence of the six subscales. This demonstrated an acceptable model fit (*χ* ^2^(804) = 2121.66, *p* < 0.001, CFI = 0.889, TLI = 0.882, RMSEA = 0.061, 90% CI [0.058, 0.064]), indicating that the six‐factor model provided a reasonable representation of the data.

### 2.3 Data Pre‐Processing

#### 2.3.1 Removal of Invalid and Incomplete Responses

Prior to finalizing the participant sample, we excluded invalid responses flagged by Qualtrics, as well as participants who completed the survey in < 500 s, who reported logical inconsistencies (e.g., indicating that their highest level of education is a Bachelor's degree, but also indicating they have completed a Master's degree), who failed two or more of four attention checks, or completed less than 70% of the CATI. These steps resulted in excluding 610 participants, mostly recruited via social media and mailing lists (excluded from Prolific 15%, Voice 58%, social media 82%, mailing lists 91%, Sona 19%, acquaintances 6%). Finally, we excluded participants with missing data for childhood or current language use, or autism diagnostic status (*n* = 86), yielding a final sample of 459 participants. No a priori power calculations were performed, but we aimed for 400–500 participants in the final sample. The final dataset can be found at https://osf.io/trbsg/overview?view_only=6dec527111024d88b0722c2ede4f95ce.

#### 2.3.2 Autism Diagnostic Status

We created a trichotomous autism diagnostic status variable from participants' self‐reports. Participants were classified as having *no diagnosis or suspicion* if they indicated no history of ASD, a negative formal assessment, or an overturned diagnosis. They were classified as *suspected* if they reported believing they may have ASD, considering or seeking diagnosis, or currently undergoing assessment. Finally, they were classified as *diagnosed* if they reported an unoverturned ASD diagnosis.

#### 2.3.3 Childhood Language Entropy

Language entropy (Gullifer and Titone 2020) reflects the extent to which multiple languages are used by or around an individual. Individuals with high entropy scores engage with multiple languages in a balanced manner. We calculated participants' childhood language entropy based on their exposure to and use of (i.e., engagement with) their different languages during childhood with the people they lived with. For each language, we computed engagement by combining the amount of use with each household member and the years lived with that person, yielding a percentage of childhood engagement per language. These percentages were entered into the *languageEntropy* function (*languageEntropy* package; Gullifer and Titone 2018) to obtain a single score per participant, with higher scores indicating more diverse language engagement.

#### 2.3.4 Current Language Entropy

A language entropy score was also calculated for current (adulthood) language engagement. We used measures of language engagement in six contexts: home, friends, extended family, work, education, and community. Entropy scores were calculated for each of these and then averaged to obtain one overall entropy score per participant. If entropy values for some contexts were missing (e.g., a participant did not work), the average was taken over the available entropy scores.

#### 2.3.5 Other Language‐Related Variables

**FIGURE 1:**
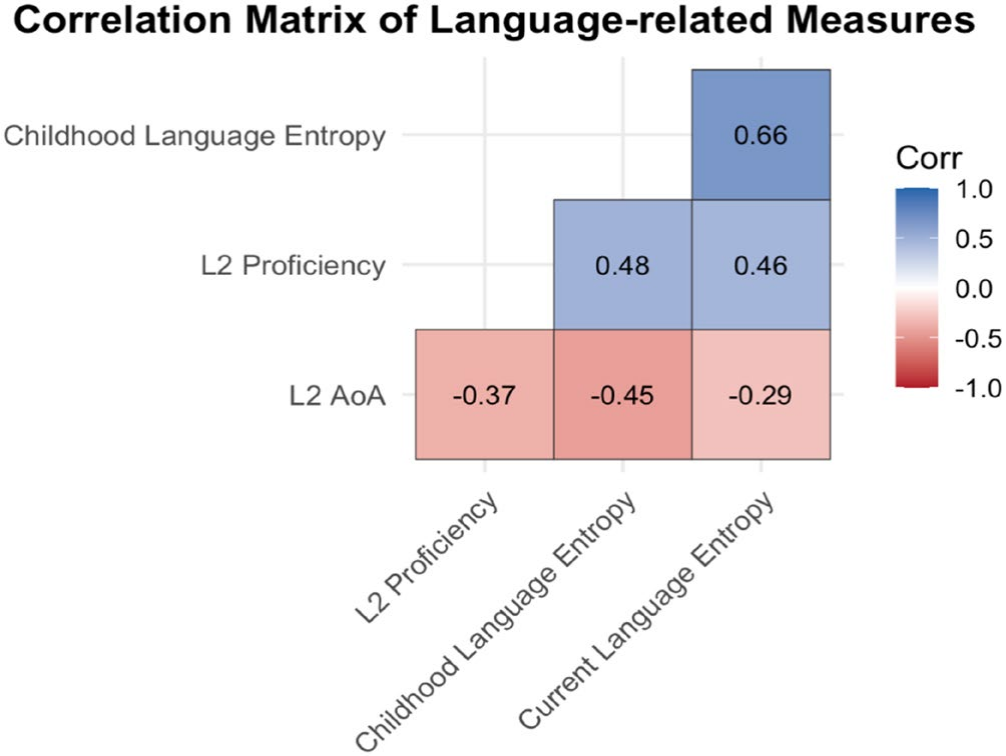
Correlation matrix of language and proficiency variables. Cell color indicates the strength and direction of Spearman correlations, with blue representing positive correlations and red representing negative correlations. Correlation coefficients are displayed inside each cell. All correlations were statistically significant (*p* < 0.001).

Finally, two additional language‐related measures were obtained: Age of Onset of L2 Acquisition (L2 AoA) in years and L2 Proficiency (how proficient one currently is in speaking their L2, on a scale of 0 “none” to 10 “perfect”). The correlations between all language‐related measures are displayed in Figure 1. Importantly, the correlation coefficients are not sufficiently strong to cause collinearity issues when adding the variables to one regression model.

### 2.4 Data Analysis

All data analyses were conducted in R (Version 4.4.1). A multiple regression methodology was employed to test effects of our two entropy measures (childhood and current language entropy) on the three social variables (CATI overall score, CATI Social Interactions, CATI Communication), leading to three linear models. We then repeated these three models with a subset of data containing only the individuals who had an autism diagnosis (*n* = 132). Finally, we were also interested in exploring the effects of L2 AoA and L2 Proficiency. This was only possible for a subset of participants who listed a second language (*n* = 338); four predictors (childhood and current language entropy, L2 AoA, L2 Proficiency) were explored in these models. All predictor variables were centered and scaled.

For each model, we used the *regsubsets* function (*leaps* package, Lumley 2024) to perform model selection and identify the best set of predictors. Model comparison was performed based on the Bayesian Information Criterion (BIC), with predictors' inclusion being warranted when they improved the model with more than two. We also included a comparison to an empty model without predictors. Partial eta squared (*ηp* ^2^) values were calculated for significant predictors using the *eta_squared* function (*effectsize* package, Ben‐Shachar et al. 2020). These *ηp* ^2^ values will be interpreted as small, medium, or large following Cohen (1969). The results are reported when significant at the 0.05‐level; however, because we are running 9 models in total, any *p*‐value above 0.0055 will be interpreted with caution (constituting a Bonferroni correction for 9 comparisons). However, all significant *p*‐values were below this threshold.

We consciously limit our analyses to those which are theoretically motivated and necessary to test our a priori hypotheses. Although the dataset's size and richness might tempt additional analyses, these would inflate Type I error rates (e.g., Barnett et al. 2022) and compromise the reliability and interpretability of the results. Researchers interested in pursuing additional, theoretically motivated analyses are encouraged to contact the first author and consult our dataset and analysis scripts at https://osf.io/trbsg/overview?view_only=6dec527111024d88b0722c2ede4f95ce.

## 3 Results

### 3.1 Descriptives

Descriptives for CATI scores and language‐related variables are presented in Table 2. On average, participants scored in the moderate range on the CATI overall (*M* = 3.35, SD = 0.81), with similar means for the Social Interactions subscale (*M* = 3.34, SD = 1.14). Measures of language entropy indicated relatively low averages, due to the number of monolingual participants in our sample. The Age of Onset of L2 Acquisition (only for the participants who spoke an L2) was around 6, with a wide range (0–44).

**TABLE 2:** Descriptive statistics for CATI scores and language‐related variables.

Variable	*N*	Mean	SD	Min	Max
CATI overall score	459	3.35	0.81	1.31	4.83
CATI social Interactions	459	3.34	1.14	1	5
CATI communication	459	2.75	1.01	1	4.86
Childhood language entropy	459	0.38	0.50	0	2.73
Current language entropy	459	0.24	0.33	0	1.88
L2 AoA	338	6.16	6.30	0	44
L2 proficiency	338	6.61	3.27	0	10

*Note:* Reported are sample size (*N*), mean, standard deviation (SD), minimum, and maximum values.

Abbreviations: CATI = Comprehensive Autistic Trait Inventory; L2 AoA = age of onset of L2 acquisition; L2 = second language.

**FIGURE 2:**
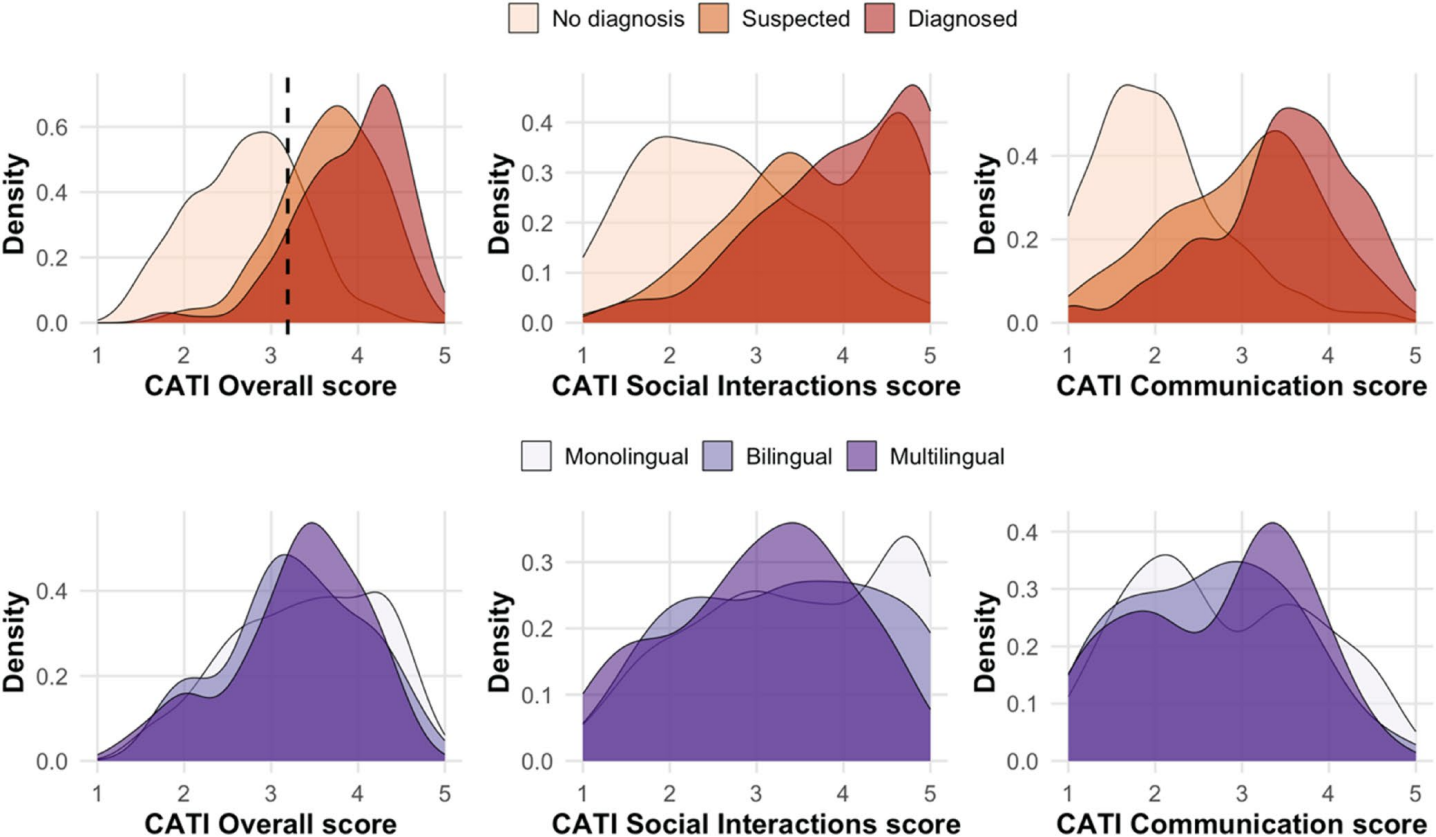
Density distributions of CATI (Comprehensive Autistic Trait Inventory) scores by diagnostic status and language background. The plots show the distribution of scores on the CATI Overall (left), Social Interactions subscale (middle), and Communication subscale (right). The top row shows these scores for participants with no diagnosis or suspicion, suspected autism, and diagnosed autism. The bottom row shows these scores for participants who self‐identified as monolingual, bilingual, and multilingual. Higher scores indicate greater presence of autistic traits. The dashed line in the top left panel represents the proposed threshold for elevated overall CATI scores based on English et al. (2021). Note that English et al. (2021) report that the proposed total score cutoff is 134. They indicate that this corresponds to a mean item response of 2.79 on the CATI five‐point Likert scale; however, since the cutoff was 134 based on 42 questions (134/42 = 3.19), it is unclear what this 2.79 is based on. We therefore plotted a line at 3.19; however, we don't use this cutoff for anything other than a visual overview.

Figure 2 presents the CATI scores grouped by diagnostic status (top row) and language background (bottom row). Participants with an autism diagnosis seem to show higher CATI overall, Social Interactions, and Communication scores (resp. means of 3.92, 3.99, 3.46) compared with those without a diagnosis (resp. means of 2.71, 2.60, 2.06), which is to be expected for a measure of autistic traits. The suspected group seems to score in between the other two groups, leaning more toward the scores of the group with an autism diagnosis (resp. means of 3.68, 3.73, 3.00). The dashed vertical line in the CATI overall score panel marks the proposed threshold for elevated overall CATI scores based on English et al. (2021), suggesting that, in our sample too, the CATI overall score seems sensitive to autism. In contrast, the distributions of CATI overall score largely overlap across self‐reported monolingual, bilingual, and multilingual participants (Figure 2, bottom row). Visually, multilinguals seem to have lower Social Interactions subscale scores compared to monolinguals, although there is substantial individual variation within each group. On the Communications subscale, this decrease in score for multilinguals compared to monolinguals is not observed.

### 3.2 Influence of Multilingualism

**FIGURE 3:**
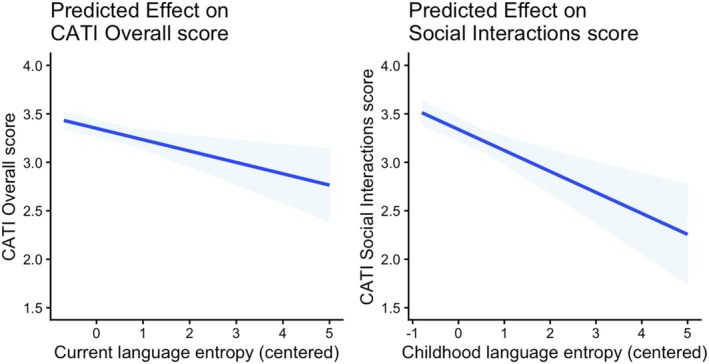
Predicted effects of current language entropy on CATI overall scores (left) and of childhood language entropy on Social Interactions subscale score (right) for the models with the total sample of 459 participants. In both cases, higher language entropy scores are associated with lower autistic traits.

We used regression analyses to test whether current and childhood language entropy predicted CATI scores (Table 3). For the CATI overall score, the best model included only current language entropy, which showed a small but significant negative effect: higher entropy was linked to lower overall autistic traits (Figure 3). For the Social Interactions subscale, childhood language entropy is the sole predictor in the best model. This effect too is negative (see Figure 3). Model *R* ^2^ values were 0.02–0.03, indicating that they explain around 2%–3% of variance in CATI scores. For the Communication subscale, neither entropy measure appeared in the best model.

**TABLE 3:** Overview of the predictors in the best models for the three different outcome measures (CATI overall, social interactions subscale, and communication subscale scores) for the total sample of 459 participants.

Outcome	Predictor	*n*	Predictor estimate	Predictor *p*	Predictor *ηp* ^2^	Adjusted *R* ^2^ model
CATI overall	Current language entropy	459	−0.12	0.001**	0.02	0.02
CATI social interactions	Childhood language entropy	459	−0.22	< 0.001***	0.04	0.03
CATI communication	—	459	—	—		—

*Note:* Childhood language entropy and current language entropy were tested as predictors. Empty cells indicate that a model without entropy predictors was best.

Abbreviation: CATI, comprehensive autistic trait inventory.

**FIGURE 4:**
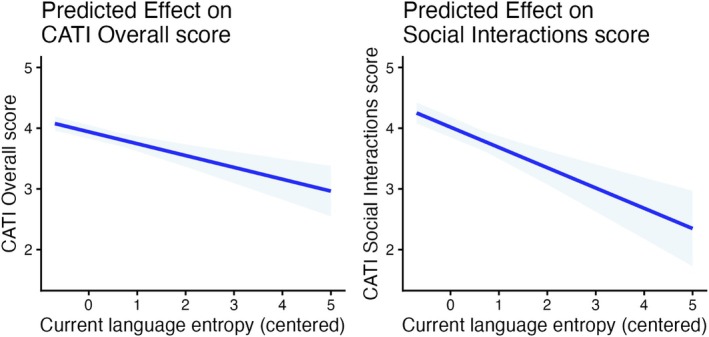
Predicted effects of current language entropy on CATI overall scores (left) and on Social Interactions subscale score (right) for the models with the subset of participants with an autism diagnosis (*n* = 132). In both cases, higher language entropy scores are associated with lower autistic traits.

We validated these relations in the subset of participants who had an autism diagnosis (Table 4). For both the CATI overall score and the Social Interactions subscale, current language entropy was the sole predictor in the best model, showing a negative effect stronger than in the full‐sample models (*ηp* ^2^ = 0.14 and 0.18 constituting large effects; see Figure 4). For the Communication subscale, the best model was empty, indicating no evidence that either entropy measure related to communication traits.

**TABLE 4:** Overview of the predictors in the best models for the three different outcome measures (CATI overall, Social Interactions subscale, and Communication subscale scores) for the subset of participants with an autism diagnosis (*n* = 132).

Outcome	Predictor	*n*	Predictor estimate	Predictor *p*	Predictor *ηp* ^2^	Adjusted *R* ^2^ model
CATI overall	Current language entropy	132	−0.20	< 0.001***	0.14	0.14
CATI social interactions	Current language entropy	132	−0.33	< 0.001***	0.18	0.17
CATI communication	—	132	—	—		—

*Note:* Childhood language entropy and current language entropy were tested as predictors. Empty cells indicate that a model without entropy predictors was best.

Abbreviation: CATI = Comprehensive Autistic Trait Inventory.

Finally, amongst participants who reported knowing a second language (*n* = 338), we examined whether L2 AoA and Proficiency, in addition to language entropy, explained variance in CATI scores. However, adding these factors did not improve any of the models. Thus, language entropy (whether in childhood or adulthood) remains the only meaningful predictor of CATI scores.

#### 3.2.1 Additional Analyses

Based on a reviewer suggestion, we further explored whether including age, gender, and SES (IMD ranking) as covariates would predict CATI scores, and importantly, would weaken the above‐reported effects of language entropy. The additional covariates, specifically age and gender, were significant predictors in several models. Importantly, however, these additional covariates did not weaken the effects of language entropy; in the full sample, the effects remained small but significant (*ηp* ^2^ = 0.02–0.04), whereas in the diagnosed sub‐sample, the effects increased in strength, from *ηp* ^2^ = 0.14–0.18 to *ηp* ^2^ = 0.16–0.21. We take this as an indication that the effects of language entropy are robust. The full output of the additional analyses is provided in S2.

## 4 Discussion

This study investigated the relation between multilingualism and social traits in adults with and without autism. Our first hypothesis (RQ1, H1) predicted that greater multilingual exposure would be associated with lower scores on the Social Interactions subscale of the CATI (English et al. 2021). This was supported by the findings: childhood language entropy showed a small but significant association with reduced social interaction traits across the full sample. In individuals with an autism diagnosis (RQ3, H3), current language entropy was associated with reduced social interaction traits. Interestingly, these effects were notably stronger than in the full sample, suggesting that multilingual exposure may have particularly pronounced benefits for social functioning in autistic populations. These findings align with the trends we observed in children (Hastedt et al. 2023; Kašćelan et al. 2019; Peristeri et al. 2020; Peristeri, Vogelzang, and Tsimpli 2021; Peristeri, Baldimtsi, et al. 2021), but contrast with studies reporting no effects (e.g., Reetzke et al. 2015). Additional analyses indicated that neither age of L2 acquisition nor proficiency improved model fit, highlighting that the diversity of language exposure appears to be the critical factor linked to reduced social autistic traits.

Our second hypothesis (RQ2, H2) predicted that multilingual exposure would also reduce overall autistic traits, driven by social interaction rather than communication abilities. Consistent with this, current language entropy predicted lower overall CATI scores in the full sample, while the Communication subscale showed no effects. This contrasts with Kašćelan et al. (2019), who only observed effects in a high‐trait subgroup. Similar effects on overall traits and absence of effects on communication were found in the autistic subgroup. The absence of an effect on communication is similar to group‐based trends with children in Peristeri et al. (2020); Peristeri, Vogelzang, and Tsimpli (2021), a Peristeri, Baldimtsi, et al. (2021).

This divergence between social interaction and communication effects warrants particular attention. Although they might sound similar, these constructs are measured separately in tools such as the ADI‐R (Lord et al. 1994), and CATI. In the CATI, social interaction reflects general social functioning and enjoyment thereof (e.g., “I generally enjoy social events” and “I find it difficult to make new friends”). In contrast, communication reflects abilities within a conversation, such as pragmatic aspects of verbal language (metaphor comprehension) and nonverbal language (reading nonverbal cues). The latter seem more constrained by linguistic competence and possibly more factual, less focussed on enjoyment and experiences. As multilingual environments naturally diversify social encounters, they may shape *interactive* behaviors without necessarily enhancing core linguistic or communicative skills. This may explain why effects of multilingualism appear for social interaction but not communication (although the latter is based on a null effect).

Although the CATI questionnaire used in this research is not a clinical tool, the observed association between multilingualism and reduced autistic traits may still carry clinical relevance. Clinicians and researchers should be aware that multilingual individuals might score differently on trait‐based measures, potentially leading to under‐identification or mischaracterization. Future research should investigate whether diagnostic tools such as the ADI‐R are sensitive to environmental and experiential factors like language diversity, which may affect some traits (i.e., social interaction) but not others (i.e., communication). It is possible that existing clinical databases (such as of the National Health Service in the UK) can be used to shed light on this, however these databases are unlikely to contain sufficiently detailed information about the patients' multilingualism. Therefore, it is likely that controlled studies in clinical settings will be needed. In addition, if multilingual exposure can meaningfully reduce social difficulties, this could inform interventions and educational practices that leverage linguistic diversity to enhance social engagement and adaptive functioning for autistic individuals.

It is of note that L2 AoA and proficiency did not explain additional variance in our models. This suggests that the *diversity* of language engagement (captured by entropy) may be more relevant than timing or proficiency in a second language. This aligns with work on the bilingual advantage, which is assumed to be driven by active use of multiple languages training attentional control (Bialystok and Craik 2022; Bialystok 2024, 2025), inhibitory skills (Green 1998), or cognitive control (Green and Abutalebi 2013) and less by proficiency.

Now that a relation between multilingual engagement and social autistic traits has been established, we can turn to the source of the observed relation. As briefly touched on in the introduction, it is still unclear whether multilingualism directly influences social skills, or whether improvements may arise, for example, via multilinguals' improved EF (Andreou et al. 2020; Peristeri, Vogelzang, and Tsimpli 2021; Romero et al. 2024) or Theory of Mind (Andreou et al. 2020; Peristeri, Baldimtsi, et al. 2021). The present study was not designed to distinguish these pathways, leaving this important question for future research.

### 4.1 Limitations

The study had several limitations. First, the questionnaires were highly verbal and asked about verbal behaviors, and as such were not suited for non‐verbal individuals. As a result, the sample represents a subpopulation of autistic individuals, limiting generalizability. Second, autistic traits, diagnostic status, and language entropy were self‐reported. Although the CATI showed good reliability, self‐reports are inherently limited by social desirability (Van de Mortel 2008) and subjective interpretation of questions. Similarly, diagnostic status was self‐reported. We did not use the trichotomous classification presented in Figure 2 (no diagnosis, suspected, diagnosed) in our analyses; however, the scores from the 3 groups are in line with English et al. (2021) and indicate that individuals who report a suspicion of autism show elevated traits. Childhood language entropy may be affected by recall bias. In addition, our UK‐based sample limits generalisability of the findings. We believe replication (in different samples) will need to take place before clinical recommendations can be made. Any replications should take into account that the CATI has been validated in several different languages/cultures, but this is, to the best of our knowledge, the first study reporting on a substantial bi/multilingual sample. Researchers should be mindful of monolingual biases and the limited availability of linguistically and culturally appropriate assessments (Leung and Molnar 2025).

Furthermore, the reader might like to know whether engaging with additional languages at a later age after having grown up monolingually might still lead to decreased social autistic traits (similar to still leading to EF improvements; Brouwer et al. 2024). Few truly childhood‐monolingual participants in our sample achieved high multilingual engagement in adulthood, so this remains untested. Future work should also explore how different types of multilingual experience and language acquisition, such as formal learning versus immersive daily use, affect social autistic traits, as our dataset lacks sufficient detail on acquisition context.

To conclude, our findings indicate that multilingualism may enhance social functioning and mitigate social interaction difficulties, especially in autistic adults. We term this the *bilingual social advantage*, distinct from the well‐known bilingual advantage in EF. Carefully characterizing this bilingual social advantage, its underlying mechanisms, and its limits will be an important task for future research, with potentially meaningful implications for supporting autistic individuals across diverse linguistic and cultural environments.

## Funding

The project was funded by an ESRC IAA Ideas to Impact Award. Sean Dong Wei Gan acknowledges internships funded by Newcastle University's Research Scholarship and Jobs On Campus.

## Conflicts of Interest

The authors declare no conflicts of interest.

## Supporting information


**Data S1.** Scoping review details.

**Data S2.** Additional analyses.
**Table A1.** Overview of the predictors in the best models for the three different outcome measures (CATI overall, Social Interactions subscale, and Communication subscale scores) for the total sample (*n* = 449). Childhood language entropy, current language entropy, age, gender, and IMD ranking were tested as predictors. CATI = Comprehensive Autistic Trait Inventory.
**Table A2.** Overview of the predictors in the best models for the three different outcome measures (CATI overall, Social Interactions subscale, and Communication subscale scores) for the subset of participants with an autism diagnosis (*n* = 129). Childhood language entropy, current language entropy, age, gender, and IMD ranking were tested as predictors. CATI = Comprehensive Autistic Trait Inventory.

## Data Availability

The dataset, analysis scripts, and Supporting Information are available at https://osf.io/trbsg/overview?view_only=6dec527111024d88b0722c2ede4f95ce.
