# Supplementary material for: A Large‐Scale Study on the Relation Between Multilingualism and Social Traits in Adults With and Without Autism Diagnoses
Source: Autism Res. 2026 Jun 23;19(8):e70300. doi: 10.1002/aur.70300 (PMC13471769; doi:10.1002/aur.70300)
Supplement: Supplementary file 1 [file AUR-19-0-s001.docx]

**Supplementary material**

***A large-scale study on the relation between multilingualism and social traits in adults with and without autism diagnoses***

**1. Scoping review methods**

A systematic search was conducted to identify studies examining the intersection between multilingualism, autism, and social interaction. Searches were performed on July 16^th^, 2024, using three major citation databases: Scopus, Web of Science, and ProQuest.

**1.1. Search strategy**

The below table contains the search strategy which incorporated three conceptual categories, each containing relevant key phrases:

| **Category** | **Key terms and phrases** |
| --- | --- |
| Multilingualism/Bilingualism | multilingual*, bilingual* |
| Autism Spectrum Condition | autism, autistic |
| Social interaction | “social interaction”, “social communication”, “social relations”, “social skills”, “nonverbal communication”, “nonverbal skills”, gestur*, “body language”, “conversational skills”, “communication skills”, “social impairment”, “socially impaired”, “social abilities”, “social responsiveness”, “social intention”, “social understanding” |

Each search required at least one term from each of the three categories to appear in the title, abstract, or keywords fields.

**1.2. Screening and selection**

The initial search retrieved **274 references**: 63 from Scopus, 133 from Web of Science, and 78 from ProQuest. Duplicates were removed using *Rayyan.ai*, leaving **161 unique citations**. Abstract screening excluded **103 papers**, resulting in **58 remaining citations.** These 58 citations were exported to Excel and combined with results from a manual search, producing a total of **67 references**. During data extraction, **7 studies** were removed due to lack of access, **12** were excluded for not including social interaction outcomes, and **9** were excluded based on publication type (five project proposals and four books). The final dataset consisted of **39 studies**, which formed the basis of the literature analysis reported in the main text. These studies are presented in Appendix A2.

**2. Scoping review output**

The below tables contain an overview of each quantitative, qualitative, and review study in the final sample of the scoping review.

**2.1. Quantitative studies**

| **Reference** | **Sample Size** | **Age (years)** | **Operationalisation multilingualism** | **Outcome Variable Type** | **Effects of Multilingualism** |
| --- | --- | --- | --- | --- | --- |
| Beauchamp et al. (2023) | 134 | Min = 10, Max = 11 | Categorical | Language skills | Positive, Neutral |
| Dai et al. (2018) | 388 | M = 2.17 | Categorical | Language | Neutral |
| Digard et al. (2020) | 289 | M = 32.4, Min = 16, Max = 80 | Continuous | Social life quality | Positive, Neutral |
| Franco & Costa (2025) | 68 parents  68 children | Children: M = 8.08, Min = 4, Max = 11 | Categorical | Social motivation and abilities, and RRBs | Neutral |
| Galicia (2021) | 30 | M = 2.75s, Min = 2.75 months, Max = 2.92 | Categorical | Social and communication skills | Neutral |
| Ge et al. (2024) | 98 | M = 6.57, Min = 4.25, Max = 9.58 | Categorical | Language | Positive, Neutral |
| Gonzalez-Barrero & Nadig (2017) | 52 | Min = 5, Max = 10. | Categorical | Language | Positive |
| Hambly & Fombonne (2014) | 33 | M = 5, Min = 3, Max = 7 | Categorical | Language | Positive, Neutral |
| Hastedt et al. (2023) | 353 | M = 2.31 | Categorical | Language and socialisation skills | Positive, Neutral |
| Iarocci et al. (2017) | 174 | Min = 6, Max = 16 | Categorical | Language (also WISC-IV) | Neutral |
| Kašćelan et al. (2019) | 394 | M = 8.3, Min = 5.3, Max = 11.4 | Categorical | Social and communication skills | Positive, Neutral |
| Li et al. (2017) | 67 | M = 8.5 | Categorical | Language; Social communication | Neutral |
| Ohashi et al. (2012) | 60 | Min = 2, Max = 4.3 | Categorical | Language, Social communication | Neutral |
| Peristeri et al. (2020) | 80 | M = 9, Min = 7.3, Max = 12 | Categorical | Language, communication, stereotypical behaviours | Neutral, Positive, Negative |
| Petersen et al. (2012) | 28 | Min = 3.6, Max = 6.1 | Categorical | Language, communication | Positive, Neutral |
| Ratto et al. (2020) | 55 | M = 4.7 | Categorical | Social communication | Neutral |
| Ratto et al. (2022) | 159 | M = 9.6 | Categorical | Social communication | Neutral, Negative |
| Reetzke et al. (2015) | 54 | M = 5 | Categorical | Social communication | Neutral |
| Siyambalapitiya et al. (2022) | 120 | M = 3.75 | Categorical | Social communication | Neutral, Negative |
| Valicenti-McDermott et al. (2019) | 462 | Min = 1, Max = 6 | Categorical | Language, Non-verbal communication, social communication | Neutral, Negative |
| Valicenti-McDermott et al. (2013) | 80 | M = 2.2 | Categorical | Language, Non-verbal communication, social communication | Positive, Neutral |
| Yow & Markman (2011) | 48 | M = 3.9, Min = 3, Max = 4.9 | Categorical | Language | Positive, Neutral |
| Zhou et al. (2019) | 37 | M = 1.75 | Categorical | Language, non-verbal communication, social communication | Neutral |

**2.2. Qualitative studies**

| **Reference** | **Sample size** | **Age (years)** | **Operationalisation multilingualism** | **Brief findings** |
| --- | --- | --- | --- | --- |
| Digard et al. (2023) | 34 | M = 10.6, Min = 3, Max = 38 | Multilingualism was defined based on families reporting the use of more than one language in the home, regardless of proficiency level. Parents indicated languages used with the child and within the broader family environment | Parents generally viewed bilingual upbringing as beneficial for family communication and cultural connection, and reported no evidence that bilingualism harmed their autistic children |
| Digard et al. (2022) | 39 | M = 33.2, Min = 16, Max = 61 | Participants self-identified as multilingual adults who spoke multiple languages (typically four or more), with multilingualism based on personal language history rather than formal proficiency testing | Multilingual autistic adults described multilingualism as enhancing self-expression, social confidence, and cultural belonging, though some experienced challenges with switching and overload |
| Nolte et al. (2021) | 54 | M = 32.7, Min = 16, Max = 64 | Autistic adults (16+) who self-identified as multilingual; most spoke ≥4 languages and used them across home, education, work, and online contexts | Participants reported cognitive strengths that supported language learning and described multilingualism as beneficial for identity, social connection, and self-efficacy, despite some communication challenges |

**2.3. Review studies**

| **Reference** | **Population** | **Operationalisation multilingualism** | **Brief findings** |
| --- | --- | --- | --- |
| Davis et al. (2022) | Mixed: autistic children, adolescents, adults, plus parents and practitioners | Exposure to or use of more than one language in home, school, or community settings, noting that definitions varied widely across included studies | No evidence that bilingualism harms autistic people and some evidence of cognitive and socio-cultural benefits; highlights *familial, clinical, and educational* barriers that reduce autistic people’s access to additional languages and calls for practice changes to ensure equal opportunities |
| Drysdale et al. (2015) | Children with ASD (and their parents) | Each study’s definitions, generally referring to children exposed to or using two or more languages at home; the review highlighted inconsistent definitions across studies | Bilingual autistic children showed similar language and social outcomes to monolinguals, with no evidence of disadvantage |
| Garrido et al. (2024) | Children with ASD (3-12 years; 169 bilingual, 159 monolingual) | Each study’s definitions, typically distinguishing children exposed to one vs. multiple languages; most studies classified bilingualism based on parent-reported exposure | No negative effects of bilingualism on language development in autistic children and possible advantages in some areas |
| Gilhuber et al. (2023) | Children with ASD (various ages across included studies) | Simultaneous or sequential exposure to two or more languages, with studies varying in whether they required comprehension, production, or mere exposure | Mostly neutral or positive effects of multilingual exposure on language and communication, with gaps in research on specific linguistic domains |
| Howard et al. (2024) | Stakeholders around autistic children (parents, practitioners, autistic individuals; focus on children but includes broader perspectives) | Based on stakeholder perspectives on children learning or using more than one language, particularly in Welsh-English contexts; exposure, use, and family background all considered | Stakeholders generally perceived bilingualism as non-harmful and beneficial, especially in bilingual education contexts such as Wales |
| Kay-Raining Bird et al. (2016) | Children with a range of developmental disorders (including ASD, developmental language disorder, Down syndrome) | Broad definition encompassing children exposed to or using more than one language in home environments; operationalisation varied by study, from minimal exposure to functional bilingualism | No evidence that bilingualism harms children with developmental disorders, including ASD. Authors advise against discouraging bilingual home environments |
| Papadopoulos et al. (2024) | Children with ASD in Greek clinical/ educational contexts | Children who used or were exposed to two or more languages in Greek educational or home settings; definitions varied across included research | No disadvantages of bilingualism for autistic children in Greek contexts, while noting the limited local evidence base |
| Prévost & Tuller (2022) | Children with ASD growing up bilingually | Encompassed exposure to, acquisition of, or use of more than one language, with a focus on bilingual language development frameworks rather than a single operational definition | Bilingualism does not negatively affect language development or autism characteristics, though individual variability is large |
| Reimann & Ratto (2023) | Families of bilingual children with ASD; focus on parents and practitioners | Families raising autistic children with more than one language, with emphasis on heritage-language use and parent language practices | Discouraging heritage-language use is not evidence-based and may harm family relationships and cultural identity |
| Sendhilnathan & Chengappa (2019) | Bilingual children with ASD in multilingual contexts (e.g. India/Singapore); focus on clinical language-intervention settings | Exposure to or use of more than one language in multilingual societies such as India; studies varied widely in proficiency and exposure criteria | Autistic children can successfully learn and communicate in multilingual contexts and restricting languages is often unnecessary |
| Trelles & Castro (2019) | Autistic children | Multilingualism was conceptualised as children having access to or being raised with more than one language, with emphasis on cultural and familial language contexts rather than strict exposure thresholds | Brief review highlighting that current evidence does *not* justify advising families to abandon bilingualism; emphasises emotional, cultural, and relational costs of restricting languages and encourages clinicians to give nuanced, family-centred recommendations |
| Uljarević et al. (2016) | Children with various neurodevelopmental conditions (including ASD, developmental language disorder, ADHD) | Exposure to or use of more than one language, noting substantial inconsistencies across studies | No evidence that multilingual exposure harms children with neurodevelopmental disorders. Encourages supportive, family-centred language practices |
| Wang et al. (2018) | Children with ASD raised in monolingual vs bilingual homes | Children being raised in homes where more than one language was spoken, with studies differing in whether they required production, comprehension, or exposure | Bilingual home environments were not associated with poorer outcomes in autistic children and were sometimes linked to modest benefits |
