# Supplementary material for: A Large‐Scale Study on the Relation Between Multilingualism and Social Traits in Adults With and Without Autism Diagnoses
Source: Autism Res. 2026 Jun 23;19(8):e70300. doi: 10.1002/aur.70300 (PMC13471769; doi:10.1002/aur.70300)
Supplement: Supplementary file 2 [file AUR-19-0-s002.docx]

**Additional analyses**

***A large-scale study on the relation between multilingualism and social traits in adults with and without autism diagnoses***

**1. Analyses including covariates of Age, Gender, and SES (IMD ranking)**

**Table A1.**

*Overview of the predictors in the best models for the three different outcome measures (CATI overall, Social Interactions subscale, and Communication subscale scores) for the total sample (n = 449). Childhood language entropy, current language entropy, age, gender, and IMD ranking were tested as predictors. CATI = Comprehensive Autistic Trait Inventory.*

| **Outcome** | **Predictor** | ***n*** | **Predictor**  **Estimate** | **Predictor *p*** | **Predictor *η*p^2^** | **Adjusted R^2^ model** |
| --- | --- | --- | --- | --- | --- | --- |
| **CATI Overall** | Current Language Entropy | 449 | -0.11 | .003 ** | .02 | .10 |
|  | Age |  | 0.13 | < .001 *** | .03 |  |
|  | Gender / nonbinary |  | 0.77 | < .001 *** | .06 |  |
|  | Gender / male |  | 0.06 | .42 |  |  |
| **CATI Social Interactions** | Current Language Entropy | 449 | -0.22 | < .001 *** | .04 | .16 |
|  | Age |  | 0.34 | < .001 *** | .09 |  |
|  | Gender / nonbinary |  | 0.97 | < .001 *** | .06 |  |
|  | Gender / male |  | 0.04 | .69 |  |  |
| **CATI Communication** | Age | 449 | 0.22 | < .001 *** | .06 | .14 |
|  | Gender / nonbinary |  | 1.09 | < .001 *** | .09 |  |
|  | Gender / male |  | 0.32 | .001 ** |  |  |

**Table A2.**

*Overview of the predictors in the best models for the three different outcome measures (CATI overall, Social Interactions subscale, and Communication subscale scores) for the subset of participants with an autism diagnosis (n = 129). Childhood language entropy, current language entropy, age, gender, and IMD ranking were tested as predictors. CATI = Comprehensive Autistic Trait Inventory.*

| **Outcome** | **Predictor** | ***n*** | **Predictor**  **Estimate** | **Predictor *p*** | **Predictor *η*p^2^** | **Adjusted R^2^ model** |
| --- | --- | --- | --- | --- | --- | --- |
| **CATI Overall** | Current Language Entropy | 129 | -0.18 | < .001 *** | .16 | .22 |
|  | Gender / nonbinary |  | 0.25 | .09 |  |  |
|  | Gender / male |  | -0.27 | .009 ** | .11 |  |
| **CATI Social Interactions** | Current Language Entropy | 129 | -0.29 | < .001 *** | .21 | .28 |
|  | Age |  | 0.32 | < .001 *** | .09 |  |
|  | Gender / nonbinary |  | 0.33 | .14 |  |  |
|  | Gender / male |  | -0.31 | .045 * | .07 |  |
| **CATI Communication** | - | 129 | - | - | - |  |

Amongst participants who reported knowing a second language (n = 338), we examined whether L2 AoA and Proficiency in addition to language entropy explained variance in CATI scores. However, adding these factors did not improve any of the models.
